# Time Course of Corticospinal Excitability and Intracortical Inhibition Just before Muscle Relaxation

**DOI:** 10.3389/fnhum.2016.00001

**Published:** 2016-01-28

**Authors:** Tomotaka Suzuki, Kenichi Sugawara, Kakuya Ogahara, Toshio Higashi

**Affiliations:** ^1^School of Rehabilitation, Kanagawa University of Human ServicesYokosuka, Japan; ^2^Unit of Rehabilitation Sciences, Nagasaki University Graduate School of Biomedical SciencesNagasaki, Japan

**Keywords:** transcranial magnetic stimulation, muscle relaxation, intracortical inhibition, motor cortex, motor evoked potential

## Abstract

Using transcranial magnetic stimulation (TMS), we investigated how short-interval intracortical inhibition (SICI) was involved with transient motor cortex (M1) excitability changes observed just before the transition from muscle contraction to muscle relaxation. Ten healthy participants performed a simultaneous relaxation task of the ipsilateral finger and foot, relaxing from 10% of their maximal voluntary contraction (MVC) force after the go signal. In the simple reaction time (RT) paradigm, single or paired TMS pulses were randomly delivered after the go signal, and motor evoked potentials (MEPs) were recorded from the right first dorsal interosseous (FDI) muscle. We analyzed the time course prior to the estimated relaxation reaction time (RRT), defined here as the onset of voluntary relaxation. SICI decreased in the 80–100 ms before RRT, and MEPs were significantly greater in amplitude in the 60–80 ms period before RRT than in the other intervals in single-pulse trials. TMS pulses did not effectively increase RRT. These results show that cortical excitability in the early stage, before muscle relaxation, plays an important role in muscle relaxation control. SICI circuits may vary between decreased and increased activation to continuously maintain muscle relaxation during or after a relaxation response. With regard to M1 excitability dynamics, we suggest that SICI also dynamically changes throughout the muscle relaxation process.

## Introduction

Muscle relaxation and muscle contraction are opposing motor actions. Nevertheless, previous studies investigating the physiology of muscle relaxation control have shown that cortical activation observed prior to voluntary muscle relaxation is similar to that observed during voluntary muscle contraction, whether measured electroencephalographically (Terada et al., 1995; Rothwell et al., 1998; Yazawa et al., 1998), with functional magnetic resonance imaging (Toma et al., 1999), or with magnetoencephalography (Toma et al., 2000). It is thus assumed that muscle relaxation is also controlled by an active cortical process similar to muscle contraction. However, how this “active” process functions is not yet clear in muscle relaxation control, because two of its main components remain unidentified: (1) the direction of the cortical activation (i.e., excitatory or inhibitory changes); and (2) the dynamicity (i.e., the number of times changes occur) of the activation.

Transcranial magnetic stimulation (TMS) studies (Buccolieri et al., 2004a; Begum et al., 2005) have shown that primary motor cortex (M1) excitability decreases prior to muscle relaxation, in contrast to muscle contraction (Starr et al., 1988; Reynolds and Ashby, 1999). We previously found that M1 was temporarily activated prior to rapid voluntary muscle relaxation, based on the time course analysis of M1 excitability changes (Suzuki et al., 2015). These changes in M1 excitability induced during muscle relaxation suggest that cortical control of muscle relaxation is established through active processing.

Intracortical inhibitory circuits within M1 play an important role in the initiation and prevention of movement, and paired-pulse TMS techniques can detect changes in both excitatory and inhibitory intracortical activity with good temporal resolution (Stinear et al., 2009). In a muscle contraction task, an increase in M1 excitability is preceded by a decrease in short-interval intracortical inhibition (SICI; Reynolds and Ashby, 1999; Soto et al., 2010). In studies employing a muscle relaxation task, SICI was reported to either increase (Buccolieri et al., 2004a) or decrease (Begum et al., 2005) prior to muscle relaxation. Although it is notable that different relaxation tasks were employed, the relationship between M1 excitability and SICI is still controversial with regard to cortical mechanisms underlying muscle relaxation.

Motawar et al. (2012) pointed out that different paired-pulse TMS techniques were used in these studies and investigated the relationship between M1 excitability and SICI during the transition from maximal muscle contraction to muscle relaxation. They found that SICI gradually increased with the progression of muscle relaxation. However, it appears likely that a critical phase in the cortical mechanism underlying quick muscle relaxation occurs before rather than after a relaxation response appears in muscle activation. Thus, we focused on this putative critical phase just before muscle relaxation. In this phase, M1 excitability does not simply decrease but instead changes dynamically. We could draw this conclusion only by analyzing the detailed time course relative to the EMG offset in each trial (Suzuki et al., 2015). However, how SICI is involved in these transient M1 excitability changes has not yet been studied.

We used TMS to investigate these relationships in the period before the transition from muscle contraction to muscle relaxation. The disparity in the relationship between M1 excitability and SICI may prevent M1 excitability changes from being detected in detail using a gross time course analysis. However, we postulate that like M1 excitability, the excitability of intracortical inhibitory circuits within M1 also dynamically changes.

## Materials and Methods

### Participants

The participants were 10 students (8 men and 2 women aged 20–35 years) from Kanagawa University of Human Services. According to a handedness questionnaire (Chapman and Chapman, 1987), nine participants were right-handed and one was cross-dominant. The mean score was 14.5, and the standard deviation (*SD*) was 2.7. None of the participants had any history of neuromuscular or physical functional impairment that might have affected task performance. All participants gave their written informed consent before the experiment in accordance with the Declaration of Helsinki. This study was conducted with the approval of the Research Ethics Committee of Kanagawa University of Human Services.

### Motor Task

The experimental design in this study generally resembled that in our previous study (Suzuki et al., 2015), in which we used a force curve measured in each trial as a reference point to analyze the time course data. Because an electromyographic (EMG) signal recorded from a target muscle is contaminated with the motor evoked potential (MEP) elicited by TMS, the EMG relaxation reaction time (RRT) cannot be detected in a trial with TMS. A force curve associated with the motor task controlled by the target muscle may be also affected by TMS. Furthermore, TMS may affect the latency of the RRT in a voluntary muscle relaxation task, given the reports indicating that TMS affects reaction time (RT) in a muscle contraction task (Day et al., 1989; Pascual-Leone et al., 1992; Palmer et al., 1994; Ziemann et al., 1997).

**Figure 1:**
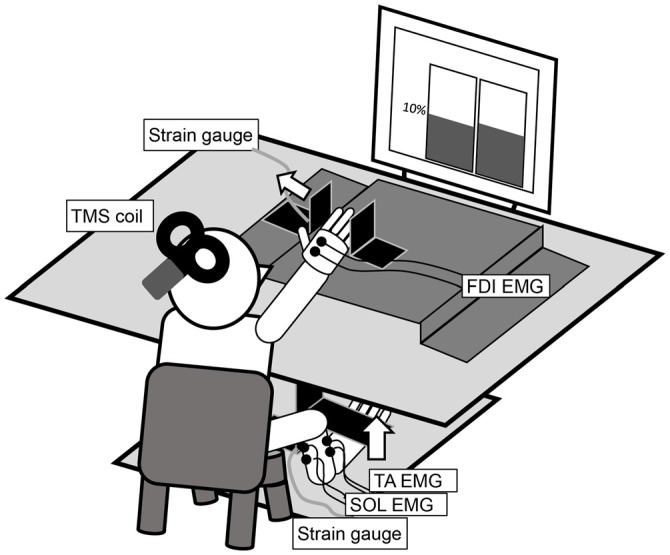
**The experimental setup for the measurement of an abduction of the right index finger and a dorsiflexion of the right ankle at 10% maximal voluntary contraction (MVC).** The left and right panels in the display in the figure are real-time representations of the abduction force in the index finger and the dorsiflexion force in the ankle, respectively.

Because of this interference, determining the RRT using data from the muscle to which TMS is delivered is exceedingly difficult. Therefore, this study utilized a motor task involving the relaxation of abduction of the right index finger and dorsiflexion of the right ankle (Figure 1). The ankle movement was chosen to eliminate contamination by MEPs. Such a motor task was reported by Kato et al. (2014), who used a simultaneous contraction or relaxation task of the ipsilateral hand and foot accompanied by isotonic contraction. Although a bilateral simultaneous relaxation task (Buccolieri et al., 2004a) was considered, we selected the unilateral simultaneous relaxation task to minimize interaction between the hemispheres.

### Experimental Paradigm

The participants sat comfortably on a chair with their right forearm pronated and digits slightly flexed on a table (Figure 1). The distal interphalangeal joint of the right index finger was positioned at the center of a hard metal plate, and the lateral surface of the little finger was fixed to another plate. The dorsum of the right foot was positioned under a cushioned metal plate. These restraints ensured finger and ankle joint motion generally did not occur. A strain gauge was mounted on the vertically bent portion of each plate. Two analog signals were amplified (SA-250 Strain Amplifier; TEAC, Tokyo, Japan and DPM-911A; Kyowa Electronic Instruments, Tokyo, Japan) and digitized (NI USB-6351; National Instruments, Austin, TX, USA). The in-progress force outputs from each strain gauge were presented in real time on a liquid crystal display monitor in front of the participant via LabVIEW (LabVIEW2009; National Instruments). The left and right panels in the display in Figure 1 present the abduction force in the index finger and the dorsiflexion force in the ankle, respectively. The target line was set at 10% of the maximal voluntary contraction (MVC), measured in advance for each motor task and individual.

At the onset of an acoustic warning signal, the participants were required to perform an isometric abduction of the right index finger and an isometric dorsiflexion of the right ankle at 10% of MVC, pressing steadily against each plate while self-controlling their performance by observing the target line. After an acoustic go signal, the participants were required to perform the transition from muscle contraction to muscle relaxation as simultaneously and quickly as possible. The interval between the warning and go signal was 3000–5000 ms, randomized with LabVIEW. The participants were instructed not to perform any voluntary movements when relaxing their agonist muscles and were particularly to avoid contracting their antagonist muscles. Before initiating the experiment, participants practiced this motor task until they were able to perform it correctly. During the practice sessions, participants received feedback from the shift of the left and right panels in the display and advice from the experimenter based on chart data.

The experiment consisted of three conditions: single-pulse TMS, paired-pulse TMS, and no TMS. Using LabVIEW and the data acquisition device (NI USB-6229 BNC; National Instruments), each TMS pulse was triggered randomly >30 ms after the go signal. We monitored the difference between the RRT and TMS pulse timing in every experimental trial and adjusted the time setting so that the TMS pulse was frequently triggered 20–100 ms before each subject’s RRT. A “no TMS” protocol was added to analyze the EMG signals and force curve data without contamination by the TMS pulse. First, “no TMS” periods of 20 trials and 210 subsequent trials was programmed so that the no TMS, single-pulse TMS, and paired-pulse TMS protocols were conducted with a probability of 10, 45, and 45%, respectively. In addition, there were 14 control trials at the beginning and end of the session; single-pulse TMS (seven trials) and paired-pulse TMS (seven trials) were delivered during isometric contraction of the target muscle at 10% of MVC.

### Measurements

The first dorsal interosseous (FDI) muscle, the agonist muscle in abduction of the index finger, was the target muscle. Surface EMGs in a belly-tendon montage were recorded from the right FDI using disposable bipolar silver/silver chloride surface electrodes (10 mm in diameter). In addition, we recorded EMG activity of the tibialis anterior (TA, agonist) and soleus (SOL, antagonist) muscles to measure the transition from isometric dorsiflexion to relaxation in the ankle. The raw signal was amplified and filtered (band pass 5–2000 Hz) with a bioelectric amplifier (Neuropack MEB-2200; Nihon Kohden, Tokyo, Japan). These EMG signals and the previously mentioned force data were digitized at 4000 Hz and stored on a laboratory computer using Power Lab system and Lab Chart 7 software (ADInstruments, Bella Vista, NSW, Australia). Additionally, the force data were filtered with a 1 kHz low pass filter.

Single-pulse and paired-pulse TMS was delivered using two Magstim 200 (Magstim, Whitland, UK) stimulators connected by a Bistim (Magstim) module and attached to a figure eight-shaped coil with an internal wing diameter of 9 cm. For single-pulse TMS, only one stimulator was triggered. The coil was placed with the handle pointing backward, laterally at 45° from the midline and approximately perpendicular to the left central sulcus, to evoke anteriorly directed current in the left hemisphere. It was optimally positioned to produce MEPs in the right FDI. Surface markings drawn on a swim cap placed on the scalp served as a reference for coil positioning. The paired-pulse TMS protocol has been shown to test SICI, elicited by a subthreshold conditioning stimulus followed by a suprathreshold test stimulus, at interstimulus intervals (ISI) of 1–6 ms (Kujirai et al., 1993). Maximum SICI is induced at ISI of 1 and 2.5 ms, suggesting that multiple mechanisms evoke SICI (Fisher et al., 2002; Roshan et al., 2003). We selected an ISI of 2.5 ms for targeting later indirect waves. The active motor threshold (aMT) was defined as the lowest stimulus intensity producing MEPs >200 μV in at least 5 of 10 successive trials during isometric contraction of the tested muscle at 10% of MVC (Rossini et al., 1994). The intensity of the test stimulus was set to 140% aMT, and then adjusted to evoke an MEP of >1 mV on average during isometric contraction of the FDI muscle at 10% of MVC. The mean aMT was 30.6% (*SD* 3.6) of the maximum stimulator output, and the mean test stimulus intensity was 144.5% (*SD* 7.6) aMT. The intensity of the conditioning stimulus was set to 70% aMT, which was reported to be able to elicit relatively pure SICI in active muscle (Ortu et al., 2008). Because SICI is reduced with increasing contraction level (Ridding et al., 1995; Ortu et al., 2008), we changed the contraction level from 20 to 10% of MVC (Suzuki et al., 2015).

### Time Course Analysis

The time course analysis prior to an RRT in each trial was necessary to identify M1 excitability changes in voluntary muscle relaxation (Suzuki et al., 2015). We expected that a simultaneous relaxation task in the ipsilateral finger and foot would enable us to identify an RRT in each trial on the basis of the EMG activity of the TA muscle, which was not influenced by TMS. However, the decline from isometric contraction at 10% of MVC to relaxation was still difficult to estimate using the EMG signal. Thus, we had to evaluate the EMG-RRT visually, as in Buccolieri et al. (2004a). Similarly, the decline of a force curve was difficult to estimate because the force curve prior to the go signal was unstable.

**Figure 2:**
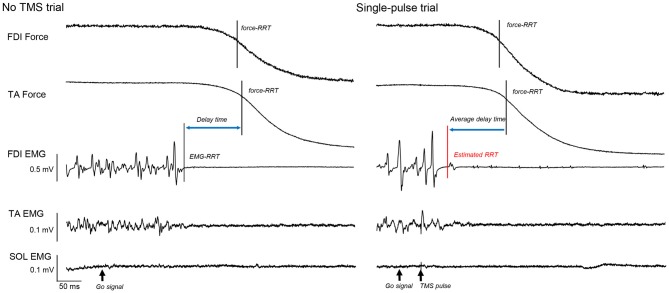
**The force curves of the first dorsal interosseous (FDI) and tibialis anterior (TA) muscles, and electromyography (EMG) of the FDI, TA, and soleus (SOL), in a “no transcranial magnetic stimulation (TMS)” and a single-pulse trial.** The solid vertical bar shows the time of the FDI and TA force relaxation reaction time (RRT). The dashed vertical bar shows the EMG-RRT of the FDI muscle. Based on this difference in “no TMS” trials, we calculated the estimated RRT from the TA force-RRT in each TMS trial (red vertical bar).

Thus, similar to our previous study (Suzuki et al., 2015), we examined the time point at which the mean of the force data in a 200 ms period before the go signal decreased to 75% of the force curve in each trial and defined it as the force-RRT. We then calculated the average time from the EMG-RRT of the FDI muscle to the TA force-RRT in the no TMS trials and subtracted this time from the time of the TA force curve 75% decrease in each TMS trial (Figure 2). We defined the time corrected in this way as the estimated RRT.

### Data Analysis and Statistics

We calculated the offline peak-to-peak amplitudes of all MEPs of the right FDI and the root mean square (RMS) value of background EMG activity of each muscle for a 20 ms period just before the TMS pulse. MEPs in TMS trials were normalized to the mean nonconditioned MEP amplitude of the control trials. FDI and TA force-RRTs were expressed as the difference between TMS and no TMS trials. Time course data of MEPs and the RMS EMG were binned into four consecutive 20 ms intervals between 20 and 100 ms before the estimated RRT (Suzuki et al., 2015), and the time course data of the force-RRTs were similarly binned relative to the average EMG-RRT in no TMS trials. These data were averaged in each bin. Additionally, we calculated SICI as the ratio between the conditioned MEPs and the nonconditioned MEPs in control and TMS trials. The data outside these time windows were excluded from analysis.

In TMS trials, to analyze a single factor (“time”) in the RRT of the FDI and TA muscle and the RMS EMG of the FDI, TA, and SOL muscle, we used one-way repeated-measures analysis of variance (ANOVA) with Bonferroni’s *post hoc* test for multiple comparisons. We also used two-way repeated-measures ANOVA to analyze two factors (“time” and “stimulus type”) in MEPs. The simple main effects of each factor were examined using *post hoc* *t*-tests and one-way repeated-measures ANOVA. We compared the difference between SICI ratios in control trials and bins using *t*-tests with the Bonferroni correction. The Spearman rank correlation coefficient was used to evaluate the correlation between the EMG-RRTs of the FDI muscle and the other RRTs in no TMS trials. All statistical analyses were conducted with IBM SPSS Statistics 20 for Windows (SPSS, Chicago, IL, USA). All statistical tests were two-tailed. Statistical significance was set at a value of *P* < 0.05.

## Results

### Relaxation Reaction Time

Table 1 provides the RRTs and the correlations between the EMG-RRTs of the FDI muscle and the other RRTs of all subjects in the no TMS trials. The time during which an EMG signal of the TA muscle decreased to the baseline level was not associated with that of the FDI muscle in “no TMS” trials. The correlation between the EMG-RRTs of the FDI and TA muscles was weaker than that between the EMG-RRTs of the FDI muscle and TA force-RRTs. Accordingly, we used the TA force-RRT to estimate the RRT in TMS trials.

**Table 1:** **Mean EMG and force RRTs (ms) in no TMS trials**.

	EMG-RRT				Force-RRT			
	FDI	TA			FDI	TA		
Subjects	*M (*SD*)*	*r_s_*	*M (SD)*	*r_s_*	*M (SD)*	*r_s_*	*M (SD)*	*r_s_*
A	154 (29)	1	170 (31)	0.86	245 (30)	0.75	252 (26)	0.82
B	175 (53)	1	189 (45)	0.86	266 (48)	0.92	292 (43)	0.94
C	138 (24)	1	180 (30)	0.46	232 (25)	0.71	250 (22)	0.58
D	174 (54)	1	186 (58)	0.63	272 (56)	0.88	292 (51)	0.79
E	154 (55)	1	189 (57)	0.73	249 (58)	0.83	286 (57)	0.91
F	168 (32)	1	204 (41)	0.46	262 (36)	0.78	281 (31)	0.77
G	132 (29)	1	145 (28)	0.83	237 (30)	0.94	267 (33)	0.76
H	149 (34)	1	176 (37)	0.71	228 (34)	0.91	262 (33)	0.82
I	181 (69)	1	220 (74)	0.72	295 (67)	0.96	301 (65)	0.9
J	151 (29)	1	275 (53)	0.05	242 (27)	0.77	271 (29)	0.64

Average	158 (17)		193 (35)		253 (21)		276 (18)

*Note: EMG, electromyography; RRT, relaxation reaction time; FDI, first dorsal interosseous; TA, tibialis anterior. The RRTs in the no TMS trials are expressed as means (SD, standard deviation). The Spearman rank correlation coefficient (r_s_) represents the correlation between the EMG-RRTs of the FDI muscle and the other RRTs*.

Table 2 shows the difference in force-RRTs between the TMS and no TMS trials in each bin. In a one-way repeated-measures ANOVA, the difference in force-RRTs between the no TMS and TMS trials demonstrated a significant main effect in both the FDI (*F*_3,24_ = 14.033, *P* < 0.001) and TA (*F*_3,24_ = 5.909, *P* = 0.004) muscles. Bonferroni’s *post hoc* test for multiple comparisons showed that FDI force-RRTs were significantly shortened in the 80–100 ms bin before the average EMG-RRT, compared with the 60–80 ms (*P* = 0.002), 40–60 ms (*P* < 0.001) and 20–40 ms (*P* = 0.006) bins, and TA force-RRTs were significantly shortened in the 80–100 ms bin compared with the 40–60 ms bin (*P* = 0.009). Only one data point was included for the 20–40 ms bin in one participant, and this was excluded from analysis.

**Table 2:** **Difference in force-RRTs (ms) between TMS and no TMS trials**.

	Time of TMS pulse relative to average RRT (0 ms)			
Muscle	−100 to −80 ms	−80 to −60 ms	−60 to −40 ms	−40 to −20 ms
FDI	−9.9 (13.4)	−1.4 (16.4)**	3.2 (14.1)**	9.5 (16.0)**
TA	−5.5 (11.3)	1.4 (13.3)	1.7 (12.3)**	8.5 (14.2)

Note: RRT, relaxation reaction time; FDI, first dorsal interosseous; TA, tibialis anterior. The force-RRTs (TMS—no TMS) are expressed as means (SD, standard deviation). **P < 0.01 compared with −100 to −80 ms.

### Changes in MEP Amplitude

**Figure 3:**
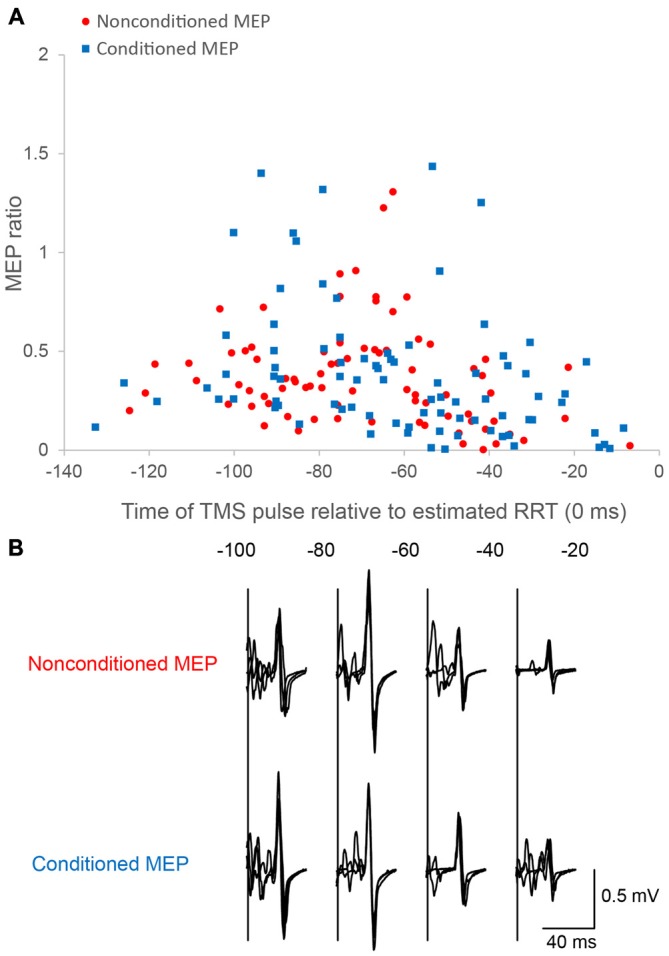
**Motor evoked potentials (MEPs) as a function of the time of the TMS pulse relative to the estimated RRT in single/paired-pulse trials.** Time course data for a single subject are plotted **(A)**. Motor evoked potential (MEP) amplitude is normalized to the mean nonconditioned MEP amplitude in control trials. The bottom panel **(B)** shows sample conditioned and nonconditioned MEP waveforms obtained from a same subject in each bin.

**Figure 4:**
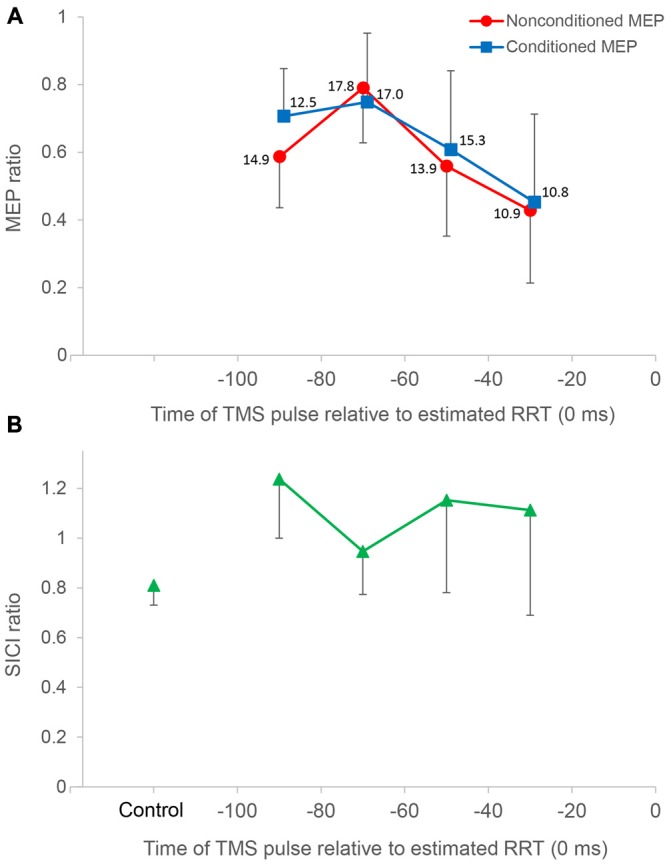
**Time course data of mean MEP amplitude and the short interval intracortical inhibition (SICI) ratio.** The top panel **(A)** shows mean MEP amplitude binned in 20 ms intervals for all subjects (*n* = 10), plotted against the time of the TMS pulse relative to the estimated relaxation reaction time (RRT; = 0 ms) in TMS trials. Average number of observations within each 20 ms period is shown beside each datum. All MEP amplitudes in single/paired-pulse trials are normalized to average MEPs in single-pulse control trials. Nonconditioned MEPs from −80 to −60 ms in single-pulse trials were significantly greater compared to those in other bins. Conditioned MEPs from −80 to −60 ms in paired-pulse trials were not significantly different from those from −100 to −80 ms. Conditioned MEPs from −100 to −80 ms were significantly greater than nonconditioned MEPs, and there was no significant difference in other bins. The bottom panel **(B)** shows the SICI ratio between conditioned MEPs and nonconditioned MEPs in control and TMS trials. The SICI ratio in the 80–100 ms period was significantly higher than that in control trials, whereas the SICI ratio in other periods was not significantly different from that in control trials. Error bars represent *SD*s.

In control trials, mean nonconditioned MEPs and conditioned MEPs were 2.52 ± 0.74 mV and 2.03 ± 0.63 mV respectively. Time course data relative to the estimated RRT showed greater MEP amplitudes in the approximately 60–80 ms period in single-pulse trials, and the 60–100 ms period in paired-pulse trials. Data from two representative cases are presented in Figure 3. A two-way repeated-measures ANOVA for the FDI MEPs showed significant interaction between “time” and “stimulus type” (Mauchly’s sphericity test, *P* = 0.676; *F*_3,27_ = 3.349, *P* = 0.034; Figure 4). A *post hoc* multiple comparison test for “time” showed that nonconditioned MEPs 60–80 ms before the estimated RRT were significantly greater than those in the 80–100 ms (*P* = 0.021), 40–60 ms (*P* = 0.003), and 20–40 ms (*P* < 0.001) periods in single-pulse trials.

**Figure 5:**
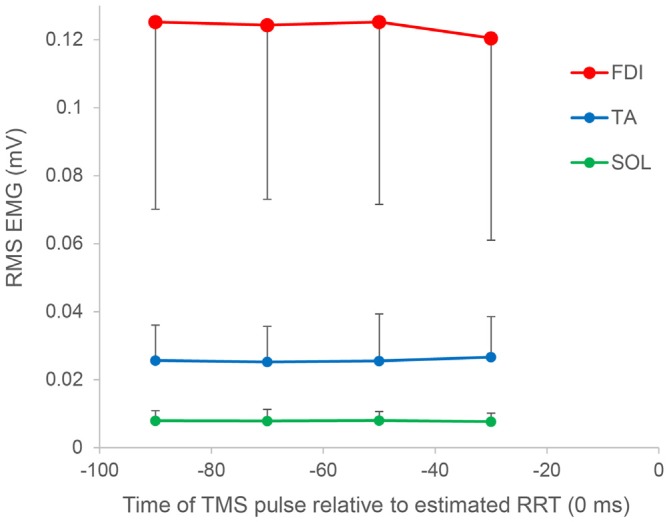
**Root mean square (RMS) electromyography of the FDI, TA, and SOL muscles as a function of the time of the TMS pulse relative to the estimated RRT.** In all muscles, no significant difference was found for any bin. Error bars represent *SD*s.

In paired-pulse trials, conditioned MEPs in the 60–80 ms period were not significantly different from those in the 80–100 ms period (*P* = 1.000) but were significantly greater than those in the 40–60 ms (*P* = 0.027) and 20–40 ms (*P* = 0.007) periods. Conditioned MEPs in the 80–100 ms period were significantly greater than those in the 20–40 ms period (*P* = 0.013). A *post hoc* *t*-test for “stimulus type” showed that conditioned MEPs were significantly greater than nonconditioned MEPs in the 80–100 ms period (*P* = 0.002), and there was no significant difference in any other bin. Furthermore, the SICI ratio between conditioned and nonconditioned MEPs in the 80–100 ms period was significantly higher than that in control trials (*P* = 0.004; Figure 4). However, the SICI ratio in other periods was not significantly different from that in control trials (60–80 ms, *P* = 0.093; 40–60 ms, *P* = 0.051; 20–40 ms, *P* = 0.170). A one-way repeated-measures ANOVA showed that no significant difference in the RMS EMG of the FDI, TA, and SOL muscle was found for any bin (*F*_3,27_ = 0.174, *P* = 0.913, *F*_3,27_ = 0.443, *P* = 0.724, *F*_3,27_ = 0.622, *P* = 0.508, respectively; Figure 5).

## Discussion

We investigated the relationship between corticospinal excitability and SICI and the effect of TMS on the RRT before muscle relaxation. Our results showed that SICI decreased significantly in the 80–100 ms period before muscle relaxation, corticospinal excitability was temporally facilitated in the 60–80 ms period before muscle relaxation, and the TMS pulse did not interfere with the relaxation response.

The findings in single-pulse trials are similar to those in our previous study (Suzuki et al., 2015), although the contraction level was lowered to investigate SICI. It has been shown that M1 excitability starts to increase approximately 80 ms before muscle contraction and increases more as it gets closer to the onset of EMG (Starr et al., 1988; Tomberg and Caramia, 1991; Leocani et al., 2000). Similarly, M1 excitability increases, rather than decreases, approximately 80 ms before muscle relaxation and subsequently decreases in reversal.

There was little extension of the force-RRT due to TMS, as estimated from the force curve data of either the FDI or TA muscle. A TMS pulse delivered just before the EMG response elongates an RT in a muscle contraction task (Day et al., 1989; Pascual-Leone et al., 1992), because focal TMS disrupts the final motor output (Ziemann et al., 1997). TMS over the motor area of a tested muscle can most effectively delay an EMG response in the homonymous muscle (Palmer et al., 1994; Ziemann et al., 1997). Begum et al. (2005) reported that a TMS pulse delivered 20–70 ms before the average RRT response extends an RRT in a muscle relaxation task. However, considering that the increase of the RT due to TMS was approximately 40 ms in previous studies (Day et al., 1989; Pascual-Leone et al., 1992; Palmer et al., 1994; Ziemann et al., 1997), our detailed time course study suggests that any lengthening effect of TMS on the RRT is minimal, even in the tested muscle. Furthermore, this work and our previous study (Suzuki et al., 2015) have shown that M1 activation specific to muscle relaxation control is induced 60–80 ms before a relaxation response (early stage), and subsequently M1 excitability drastically decreases toward the termination of muscle relaxation (later stage). Therefore, we propose that the TMS pulse should not affect muscle relaxation control, since M1 activation in the later stage is a less active process.

If a TMS pulse was delivered 80–100 ms before muscle relaxation, both the FDI and TA had slightly shorter RRTs. This shortening effect of TMS on RRTs may be partly caused by intersensory facilitation. Although the TMS pulse is triggered >30 ms after the go signal in this study, intersensory facilitation is assumed when a slight shortening effect on RT remains if a second stimulus is presented up to 50 ms after a first stimulus (Nickerson, 1973; Ziemann et al., 1997).

A decrease in SICI preceded a temporal increase in M1 excitability. Subsequently, although the magnitude of SICI was reduced, SICI was still decreased compared to the period during sustained contraction at 10% of MVC. This result is somewhat consistent with Begum et al. (2005), who reported that SICI decreases in the interval of 20–70 ms before muscle relaxation. However, their results showed that M1 excitability also decreases before muscle relaxation. For the first time, we have shown a clear case in which increased M1 excitability preceded decreased SICI. Active changes in M1 excitability may be related to the hypothesis that corticospinal neurons activate spinal inhibitory neurons in motor inhibition during muscle relaxation (Begum et al., 2005) and that the decrease in reflex excitability during a muscle relaxation task is mainly mediated by presynaptic inhibition, contributing to early motor neuron de-recruitment (Schieppati and Crenna, 1985). However, it has not been shown that the excitability of the Hoffmann reflex is significantly modulated in spinal mechanisms in the early stage before muscle relaxation. Thus, we believe that temporal facilitation of M1 excitability alters the excitability of spinal motor neurons, resulting in subsequent reduced cortical activation and a decline in an EMG response.

We expected that SICI would be changed from decreasing to increasing prior to muscle relaxation. However, an increase in SICI was not observed in the 20–100 ms period before an RRT. Motawar et al. (2012) showed that SICI gradually increases with the progression of muscle relaxation, i.e., after a relaxation response. The magnetoencephalography study by Toma et al. (2000) suggested that 20 Hz synchronization measured after muscle relaxation is related to the deactivated cortical areas, and this deactivation may have already started around the time of the relaxation response. Taken together with these findings, we hypothesize that SICI circuits are changed from being decreased to being increased around or after an EMG response caused by muscle contraction attenuation (after stage). A decrease in M1 excitability with strong SICI is also observed after the no-go signal in a go/no-go reaction task (Hoshiyama et al., 1997; Sohn et al., 2002) and when preventing unwanted muscle activation (Stinear and Byblow, 2003). In this study, reduced SICI in the early stage suggests that the inhibitory circuits are disinhibited by input from interneurons, so the disinhibition may facilitate M1 excitability. In addition, strong SICI is not induced if the relevant muscle continues to contract, instead seeming to continuously suppress the muscle activation at rest or under decline. Likewise, at the spinal level, the excitability of the Hoffmann reflex is strikingly reduced after muscle relaxation (Schieppati and Crenna, 1984) and shows the first signs of being slightly depressed approximately 20 ms prior to muscle relaxation (Schieppati et al., 1986).

Not only M1, but also bilateral supplementary motor areas, are activated during muscle relaxation control. We believe that M1 and SICI changes in the early-stage before muscle relaxation are not related to spinal-level changes. However, since this study only investigated excitability changes in M1, it remains unclear how other motor-related areas affect the excitability of spinal motor neurons.

In our time course study, the nonconditioned MEP size was not adjusted because our experimental protocol requires that a large number of trials be conducted for the different conditions for which the test stimulus intensity is adjusted. The nonconditioned MEP size may influence the magnitude of SICI (Sanger et al., 2001; Daskalakis et al., 2002). If the time course of SICI modulation is investigated in not only the “before” but also the “around” or “after” phases of the relaxation response, the test stimulus intensity should be adjusted for the remarkably decreased MEP amplitude. Additionally, because this testing would occur under resting conditions, when background EMG activity had returned to baseline, the conditioning stimulus intensity must also be changed.

SICI decreased in advance of increased M1 excitability. However, it was possible that short-interval intracortical facilitation (SICF) had increased, given that the SICI ratio in experimental trials approximated 1. Subthreshold TMS is able to activate both inhibitory and excitatory cortical interneurons, and the final output of the corticospinal neuron is influenced by both circuits (Ziemann et al., 1996; Ortu et al., 2008). We selected a conditioning stimulus intensity at which SICI was activated in the absence of SICF (Ortu et al., 2008). However, in most studies, paired pulse TMS has been delivered when intracortical circuits are likely to have reached a steady state, i.e., at rest or during sustained contractions (Floeter and Rothwell, 1999). Therefore, it remains unclear how the SICI and SICF circuits are involved in cortical activation while still in a dynamic state during muscle relaxation control.

Taken together with previous studies (Schieppati and Crenna, 1984; Toma et al., 2000; Motawar et al., 2012), our results indicate that SICI circuits may switch from being decreased to being increased as time progresses towards the after stage, thus maintaining muscle relaxation after an RRT. We suggest that SICI related to M1 excitability also dynamically changes during the development of muscle relaxation control (Suzuki et al., 2015). This may partly explain the disparity regarding SICI identified in previous studies (Buccolieri et al., 2004a; Begum et al., 2005).

## Conclusion

SICI was remarkably decreased in the 80–100 ms before rapid muscle relaxation, and this was followed immediately by a temporary activation of M1, which was deactivated thereafter. We conclude that cortical activation in this early stage plays an important role in muscle relaxation control. In the later stage, unlike in muscle contraction, TMS has little lengthening effect on the RRT. A longer RRT is reported in patients with impaired inhibitory control, such as in dystonia (Buccolieri et al., 2004b) and stroke (Chae et al., 2002). If it is assumed that cortical activation in the early stage of relaxation control promotes smooth muscle relaxation, it is possible that an intervention to facilitate this effect would be effective in lessening a longer RRT.

This study supports the hypothesis that muscle relaxation control is established through active processing, similar to muscle contraction. Furthermore, we propose that muscle relaxation requires more complex motor control than muscle contraction. In a recent study, Sugawara et al. (2016) reported that SICI is specifically modified during muscle relaxation in motor learning. Appropriate muscle relaxation control is a prerequisite for fine motor skills. Improved motor function could then result from the increased attention to muscle relaxation as well as contraction related to the frequent repetition of muscle contraction and relaxation.

## Author Contributions

TS, KS, and TH conception and design of research; TS, KS, and KO acquisited the data; TS and KO analyzed data; TS, KS, KO, and TH interpreted results; TS drafted manuscript; TS, KS, and TH revised manuscript; TS, KS, KO, and TH approved the version to be published.

## Funding

This work was supported by JSPS KAKENHI Grant Number 25750212.

## Conflict of Interest Statement

The authors declare that the research was conducted in the absence of any commercial or financial relationships that could be construed as a potential conflict of interest.
